# Puerperal Eclampsia*Read at Palestine Meeting of the East Texas Medico-Chirurgical Society, May 29, 1902.

**Published:** 1903-03

**Authors:** E. B. Parsons

**Affiliations:** Palestine, Texas


For Texas Medical Journal.

### Puerperal Eclampsia.*

BY E. B. PARSONS, M. D., PALESTINE, TEXAS.

Puerperal eclampsia, as commonly used, is synonym for puerperal convulsions, which is characterized by a series of convulsive movements of the body with loss of consciousness and coma; coma following it, and it may occur during pregnancy, labor or child-bed. Eclampsia is not exceedingly rare, and its dangers are great for both mother and child, for if the mother escape death, she is liable to septic infection, nephritis, psycho-motor and psycho-sensorial sequlæ. Its importance is pre-eminent, since the etiology is in doubt; hence, the difference of opinion among authors as to its medical and obstetrical management. As to its relative frequency it occurs in about one to every two hundred and sixty cases. The periods of time when eclampsia occur has been studied by Goldberg and others. In eleven hundred and twenty cases, twenty-one and seven-tenths occur during pregnancy. Fifty-six and thirty-four one-hundredths per cent. during labor, and after labor twenty-two and fifty-nine one-hundredths per cent.

---

*Read at Palestine Meeting of the East Texas Medico-Chirurgical Society, May 29, 1902.

*Premonitory Symptoms.*—While there are a great many, I shall only call your attention to three of the most prominent ones, viz.: Epigastric pains, headache, disturbance of vision. Should these occur in a pregnant woman who is œdemitous, scanty urine, diminished amount of urea with albumen and casts, we may be pretty sure of a case of puerperal eclampsia, unless prevented. With these brief descriptive remarks, I will pass on to more important and essential causes, with a brief mention of the predisposing and exciting causes, in so far as plura parity and prima parity reflect excitability. In regard to prima parity, it occurs from three to seven times more frequently from greater intra-abdominal pressure, excessive nervous excitability, long labors of a primapara, and, if she be old, increased dangers.

### PLURA PARITY.

Here we have the same causes as the former, with twice the work thrown on the eliminating organs. Also they are more liable between the ages of twenty and thirty, which revert back to prima parity.

### REFLEX.

Any reckless handling of the patient, dilating the os, pressure over abdomen, uterine contraction, distended bladder and rectum. We pass on now to essential causes. The student who will ponder over the present literature on this subject will readily conclude that the patho-genesis of puerperal eclampsia is by no means perfectly clear. We have various theories by different authors; some of the most prominent being the nephritic origin, which, I would rather conclude, was more the result than an initiatory to the cause.

### THE MICROBIAN THEORY.

Of which great doubt exists, and is positively denied, as we find the same micro-organism in pregnant women who do not have eclampsia. In order to be as brief as possible, the one cause generally accepted by most writers today is toxemia, and from whence is derived auto-intoxication. Every symptom of the disease proves beyond a doubt its auto-toxic origin. This toxin absorbed cripples all of the excretory organs, bowels, liver, skin, lungs and kidneys, inducing in them a state of stasis and consequent defective elimination. The blood becomes charged with poison, resulting from defective metabolism. The pabulum of the nerves has been altered until tired nature refuses to act, and a break-down ensues. Figuratively speaking, the band on the fly-wheel breaks, the governor drops, hence the machinery runs away with itself.

E. P. Davis, of Philadelphia, says, "An effort to positively identify eclampsia with uremia has failed, for many uremics die without eclampsia, and many of the eclamptics do not present the pathological signs of uremia." W. A. N. Dorland claims that from the great constancy of hepatic lesions, necrotic and hemoratic, that have been noted in autopsies upon eclamptic women and the accompanying urinary changes indicative of imperfect katabolism have inclined the concensus of opinion towards the auto-intoxication in eclampsia with the greatest interest, centering upon the liver, as the probable laboratory where the poison is engendered. The liver, you will note, has a triple rôle to perform. It is the chemical laboratory of the body; collects certain toxic principles and converts them into the blood to excrete the bile, and transforms the other poisons in a similar manner, and through antiseptic properties reduces intestinal fermentation. We all know that pregnancy favors organic insufficiency. The waste materials are increased; also an increase in the production of leucomaines, and of the organs of defense—the intestinal tube, spleen, lymphatic glands, super-renal capsules and liver, etc., plus the eliminating organs, intestines, skin, lungs and kidneys. If these be altered by intoxication, and not in perfect accord, and perfect metabolism under the constant and predisposing condition of pregnancy, toxicity will result. From a practical standpoint, I think the liver plays an important rôle in the production of eclampsia, for I have succeeded in relieving what I am almost sure would have been convulsions by the timely administration of hepatic stimulants and sedatives, viz.: calomel, soda, etc.

TREATMENT.

The great dissimilarity among physicians in the treatment of this affection teaches us an important lesson, and that is, that there is no routine treatment which will fullfil all indications in all cases, for if we understood more fully the peculiar poison-producing eclampsia, in each particular case, we could understand why such and such particular treatment is successful in a given case. In all cases we must employ diuretics, diaphoretics, purgatives and sedatives; croton oil drops too, emulsified in eclasia or elaterium grains, one-quarter placed on back of tongue and gently washed down with milk or water, usually suffice for a purgative, but this may be followed by a compound jalap powder, saturated solution of magnesia sulphate and a mild chloride of mercury. As a diaphoretic and diuretic, I think hypodermoclysis and entroclysis of normal saline solution is first, hot baths, vapor baths, hot air baths, etc. As to sedation, chloroform stands first on the list; chloral morphia, verat vir., venesection—each may be used according to the varying indication. As to morphia, I never use, if possible, owing to its effect on secretion, and if nephritis exists, it would do much harm. The only time it is admissible is when the protein in the urine consists of serum globularim and not serum albumen, or an excess of the globulum, which may be determined by chemical analysis. Chloral is a much better drug used to control convulsions. For instance, an injection of fifteen grains of chloral dissolved in sweet milk every fifteen minutes until sixty grains or more are given. The treatment in all cases should be begun with the inhalation of chloroform. Verat vir. is extremely used, and more especially in the South. It is a spinal cardiac depressive. It impairs the reflexes, but does not affect sensation. It produces motor-paralysis centrally. With this physiological action we would of course expect to allay convulsions, or any motor disturbance. In my experience, it acts favorably in selected cases, but must be given heroically, from five to twenty drops hypodermically every hour. Reduce the pulse to sixty, and hold at that rate, if any good is to be accomplished, but it must be watched, as it reduces excitability of the nerve centers, like morphia or chloral, without removing the cause.

*Venesection.*—In selected cases, I believe this to be the ideal treatment, but like many other good agents, it is potent for evil as well as good. An eclamptic who is plethoric should be bled and simultaneously given normal saline solution by venesection or hypodermo or interero clysis. Literally flood the tissues with this excretory agent. We replace the blood of venesection, we dilute the remaining toxics, we stimulate all of the excretory organs to renewed action, thereby getting rid of the poison in the most natural and feasible way. If my patient be extremely anemic, omit venesection, but use normal solution freely. To dilute toxin and eliminate them: Produce sedation by any of the various means I have mentioned, and empty uterus by dilating os instrumentally sufficient to get two fingers in, proceed to dilate manually until the blade of your faucet will enter. If head is not well down, produce version, and if breech, produce version. After delivery, if patient be in a stupor, stimulate and follow up the treatment with saturated solution of sulphate of magnesia as a further aid at elimination by catharsis. It is exceedingly difficult in a disease so complex as this to lay down specific formulæ to be governed by, but each case must be treated upon its own merits—a treatment that seems to me to be applicable to the majority of cases. I will assume that our case is rather plethoric and the convulsions have begun. I administer chloroform, and as soon as the convulsions have worn off, I commence to bleed, at the same time give normal saline solution either by hypodermo or entroclysis or intravenously. Place two drops of croton oil on the tongue, envelope patient in a blanket that has been dipped in hot water and rung out dry, put dry covering over this, pour hot alcohol over hot bricks at the feet, thereby giving vapor bath. If convulsions recur, give from twenty to sixty grains of chloral hydrate by rectum, and inject five to ten drops of Norwood's tincture of verat virdi. Repeat as necessary chloral and verat vir. Stimulate if necessary. Empty uterus as soon as practicable. This, I think, comprises the present status of opinion on eclampsia of today.

